# Carriage and potential long distance transmission of *Listeria monocytogenes* by migratory black-headed gulls in Dianchi Lake, Kunming

**DOI:** 10.1080/22221751.2019.1647764

**Published:** 2019-08-08

**Authors:** Lin Gan, Xiaolong Cao, Yan Wang, Yiqian Wang, Huaying Jiang, Ruiting Lan, Jianguo Xu, Changyun Ye

**Affiliations:** a State Key Laboratory for Infectious Disease Prevention and Control, National Institute for Communicable Disease Control and Prevention, Beijing, People’s Republic of China; b Beijing Changping Institute for Tuberculosis Prevention and Treatment, Beijing, China; c Guizhou Medical University, Guiyang, China; d University of New South Wales, Sydney, Australia

**Keywords:** *Listeria monocytogenes*, black-headed gull, ST, transmission, migration

## Abstract

*Listeria monocytogenes* is a high risk pathogen which can cause invasive diseases in humans. We previously reported that black-headed gulls from Dianchi Lake of Kunming carrying *L. monocytogenes*, while the characteristics of these isolates and the relationship with habitats of migratory gulls have not been explored. In this study, we investigated the prevalence and molecular characteristics of *Listeria monocytogenes* from black-headed gulls in Dianchi Lake, and phylogenetic analysis based on core genome SNPs was used to determine the genetic relationship of the strains from Dianchi Lake and other regions. Occurrence of *L. monocytogenes* in black-headed gull feces in 2016, 2017 and 2018 was 1.0%, 1.0% and 0.6% respectively. The predominant serotype of 28 isolates was 4b, while the predominant sequence types were ST145 and ST201. Based on their prevalence and genomic relationships, ST5 and ST87 were likely to be sourced locally while ST145 and ST201 were likely to be non-local. *L. monocytogenes* may travel along the bird migration route leading to transmission over a large geographical span carried by black-headed gull. Although the prevalence of *L. monocytogenes* was low, its carriage by the migratory black-headed gulls poses potential public health risks in regions where the migratory birds passage and reside.

## Introduction

*Listeria monocytogenes* is a Gram-positive bacterium which can cause food-borne diseases in humans. It is an intracellular bacterium and has the ability to penetrate the intestinal, blood–brain and placental barriers, and cause a series of clinical conditions such as diarrhea, septicemia, meningoencephalitis and miscarriage [1,2]. *L. monocytogenes* is also a multihost bacterium which can cause listeriosis in many domestic animals and wild animals including cows, goats, wild rodents and wild birds with similar pathological manifestations [3–9]. *L. monocytogenes* can cause listeriosis in sporadic cases and outbreaks. Listeriosis has a high mortality rate, and in most cases, immunocompromised, debilitated individuals are especially susceptible to *L. monocytogenes*. The recent outbreak caused by ready-to-eat (RTE) foods in South Africa had a mortality rate of 28.6%, and the most affected age group was the neonates ≤28 days [10].

Of the 13 *L. monocytogenes* serotypes, serotype 1/2a, 1/2b and 4b account for most of the human clinical cases, and serotype 4b is responsible for the majority of outbreaks [11]. Most of the key virulence-associated genes are distributed in pathogenicity island 1 (LIPI-1), LIPI-3 and LIPI-4. LIPI-1 is widely distributed in most *L. monocytogenes* strains, and harbours several important virulence genes, such as the central virulence regulator *prfA*, the surface protein necessary for actin assembly *actA* and the phospholipase gene *plcA*. LIPI-3 encodes listeriolysin S (LLS), which reinforce the cytotoxic and hemolytic activity of *L. monocytogenes*, and is only harboured by some lineage I strains [12]. LIPI-4 encodes a cellobiose-family phosphotransferase system (PTS) which is present in CC4 and CC87 *L. monocytogenes*, and contributes to placental and central nervous infections [13,14].

Black-headed gull (*Larus ridibundus*) is a widely distributed migratory wild bird that has been shown to carry several pathogens such as *Influenza A virus, Escherichia coli*, *Salmonella* and potentially pathogenic microfungi [8,15–18]. In China, black-headed gulls migrate to Kunming by way of Siberia, Mongolia, Neimenggu, Xinjiang, Ningxia, Qinghai, Gansu, Shaanxi and Sichuan every year (November to March). In 2016, we isolated *L. monocytogenes* from black-headed gull feces in Dianchi Lake of Kunming, southwestern China, and found the isolates had potential infection risk for residents and tourists in Kunming [19]. However, the genomic characteristics of *L. monocytogenes* from black-headed gulls and the relationship between Dianchi Lake isolates and strains from other sources (Kunming and other geographical sites) have not been explored. In this study, we investigated the longitudinal prevalence and molecular characteristics of *L. monocytogenes* from black-headed gulls in Dianchi Lake and food samples from nearby retail markets. Serotyping, multilocus sequence typing (MLST) and pulsed-field gel electrophoresis (PFGE) were utilized to identify the diversity of *L. monocytogenes* isolates, and whole genome sequencing (WGS) was utilized to analyze the genetic relationship of strains from diverse origins.

## Materials and methods

### Sample collection

Black-headed gull fecal samples and environmental swabs of Dianchi Lake were randomly collected in Jan 2017, Nov 2017 and Jan 2018. A total of 2135 black-headed gull fecal samples and 300 environmental swabs were collected. All fecal samples were collected on the beach ground of Dianchi Lake as soon as they were excreted from Black-headed Gull. Feces were collected at a distance of at least 5 m from each other, to avoid collecting samples from the same gull. A total of 388 food samples from 80 booths and 4 restaurants of retail markets which are distributed in three districts proximal to Dianchi Lake, were collected in Nov 2017 and Jan 2018. Samples included pork, beef, frozen meat, mutton, chicken, duck, fish, ready to eat (RTE) foods, and environmental swabs on refrigerators, tables, knives and meat trays.

The fresh feces and environmental swabs were stored in 5 mL brain heart infusion (BHI) broth containing 20% glycerol by using sterile cotton swabs, food samples were obtained with sterile bags, and stored at 4°C before isolation in the laboratory.

### Isolation and identification

*Listeria* was isolated according to ISO 11290 method with partial modifications. Isolation was divided into two step enrichments. For fecal samples, about 2–5 g of feces were collected into 10 mL Half Fraser broth; for environmental swabs, 1 mL of samples were collected into 9 mL Half Fraser broth; for food samples, 25 g of sample was added to 225 mL Half Fraser broth, and incubated at 30°C for 24 h. Then 1 mL of primary enrichment cultures were added to 5 mL Fraser broth and incubated at 37°C for 48 h. Two loopful of secondary enrichment broths were steaked on the Chromogenic *Listeria* Agar (Oxoid, Basingstoke, UK), incubated at 37°C for 48 h. Five presumptive positive colonies were selected for each samples, and each colony was identified by PCR for *Listeria* genus-specific gene *prs* and species-specific gene *lmo0733*. Primers were shown in Table S1.

### Serotyping, MLST and PFGE analysis

*L. monocytogenes* isolates were serotyped by multiplex PCR assay and antigen serum agglutination assay (Denka Seiken, Japan) [20]. Multilocus sequence typing (MLST) analysis was performed by sequencing seven housekeeping genes. Housekeeping genes included *abcZ*, *blgA*, *cat*, *dapE*, *dat*, *ldh*, *lhkA*, and primers are shown in Table S1. Through submitting sequences to the Institute Pasteur *L. monocytogenes* MLST database (http://bigsdb.pasteur.fr/listeria/listeria.html), ST type of each isolates were confirmed [21,22]. For PFGE analysis, *AscI* restriction enzymes were used to subtyping according to the PulseNet protocol of *L. monocytogenes* in the Chinese Centers for Disease Control and Prevention [23].

### Whole genome sequencing

The 29 isolates from Dianchi Lake 10 representative market-source isolates which have the same sequence types or pulsotypes with isolates from the Black-headed gulls, ICDC-LM497 (ST14, isolated from food in Xinjiang province, preserved in the China CDC) and ICDC-LM2875 (ST145, isolated from human in Hebei province, preserved in the China CDC) were selected for sequencing (Table S2). Selected isolates were cultured in brain–heart infusion broth (BHI) at 37°C overnight. DNA was extracted by Wizard® Genomic DNA Purification Kit according to the manufacturer’s introduction. Purified DNA was detected by agarose gel electrophoresis and quantified by Qubit. Whole-genome sequencing was performed on the Illumina Hiseq PE150 platform by Novogene (Beijing, China) using *L. monocytogenes* EGD-e as the reference strain, and the genome sequences were assembled into a number of scaffolds by SOAP denovo (version 2.04).

### SNP-based phylogenetic analysis

Sequenced strains and other strains with genome sequences available online sharing the same sequence type with isolates from Dianchi Lake (except strains from unknown region) were chosen for comparison (Table S2). SNPs were detected through comparisons of *L. monocytogenes* genomes using MuMer, and each SNP was not positioned at a recombination region and the distance between two SNP sites ≥5 reads. The maximum likelihood trees were constructed by MEGA7 based on the core genome SNPs. Tamura-Nei model was used, and bootstraps were performed with 1,000 replicates.

### Identification of virulence genes

For virulence identification, the 29 isolates from Dianchi Lake were analyzed on website of Center for Genomic Epidemiology (https://cge.cbs.dtu.dk/services/VirulenceFinder/), and the analysis were performed with the minimum of 80% length and 85% identity [24].

## Results

### Occurrence and molecular subtyping of *L. monocytogenes* isolates from Dianchi Lake black-headed gulls

Sampling was conducted for 3 years from 2016 to 2018. Data of 2016 was previously published and were included in this study for completeness [19]. A total of 3030 black-headed gull feces and 300 environmental swab samples from Dianchi Lake were screened for *L. monocytogenes* in the three consecutive years. Occurrences of *L. monocytogenes* in black-headed gull feces in 2016, 2017 and 2018 were 1.0% (9/895), 1.0% (16/1612) and 0.6% (3/523) per year respectively, and only one isolate from Dianchi Lake environment was found in 2017 (0.7%, 1/150) and none in other years. The 29 *L. monocytogenes* isolates from Dianchi Lake were subtyped into 5 serotypes by serotyping, 9 sequence types by MLST and 11 pulsotypes by PFGE. The predominant serotype was 4b (51.9%), while the predominant sequence types were ST145 and ST201, accounted for 41.8% and 17.2% of isolates respectively. ST3, ST5, ST145 and ST201 were found in two different years (Table 1, Figure S1).

**Table 1.** Prevalence and subtyping characteristics of L. monocytogenes (LM) in Dianchi lak**e.**

Sample types	District	Time	No. of samples (No. of LM)	Sequence types	Pulsotypes
Black-headed gull feces			**3030** (**28)**	** **	** **
	Dianchi Lake	Mar 2016	895 (9)	ST3^b^, ST5^b^, ST35, ST201^b^	PT22, PT51^b^, PT59, PT351
	Dianchi Lake	Jan 2017	950 (16)	ST3^b^, ST14, ST73, ST145^a,b^, ST201^b^	PT23, PT33^a,b^, PT51^b^, PT261, PT374
	Dianchi Lake	Nov 2017	662 (0)	–	–
	Dianchi Lake	Jan 2018	523 (3)	ST5^b^, ST87, ST145^b^	PT30, PT33^b^, PT369
Environmental swabs	** **	** **	**300** (**1)**	**–**	**–**
	Dianchi Lake	Jan 2017	150 (1)	ST2	PT34
	Dianchi Lake	Nov 2017	72 (0)	**–**	**–**
	Dianchi Lake	Jan 2018	78 (0)	**–**	**–**
Food samples	** **	** **	**388** (**64)**	** **	** **
	Retail Markets	Nov 2017	213 (30)	ST2^b^, ST3^b^, ST8, ST9^b^, ST37, ST87^a,b^, ST121^b^, ST155, ST330, ST391	PT4^a,b^, PT9^b^, PT11, PT12, PT16^b^, PT27, PT30^b^, PT32, PT34^b^, PT37, PT178, PT310, PT318^b^, PT371, PT372
	Retail Markets	Jan 2018	175 (34)	ST2^b^, ST3^b^, ST5, ST7, ST9^b^, ST87^a,b^, ST101, ST121^b^, ST288	PT4^b^, PT9^b^, PT16^b^, PT17, PT22, PT26, PT30^a,b^, PT34^b^, PT36, PT45, PT52, PT53, PT263, PT317, PT318^b^, PT373

^a^Predominant subtype in isolates.

^b^Subtype which collected ≥ two times in different years.

To determine the prevalence and genotypes of *L. monocytogenes* in the local retail markets, 388 food samples and environmental swabs were collected from three retail markets near Dianchi Lake and 64 samples were positive for *L. monocytogenes* (16.49%). The 64 isolates were divided into 4 serotypes, 14 sequence types and 25 pulsotypes. The predominant serotype and sequence type were 1/2b (42.2%) and ST87 (28.1%) respectively, and the subtypes shared between Dianchi Lake and retail markets were ST2 (PT34), ST3, ST5 (PT22) and ST87 (PT30) (Table 1, Figure S1).

### Genomic relationships of *L. monocytogenes* isolates from Dianchi Lake black-headed gulls

**Figure 1.**
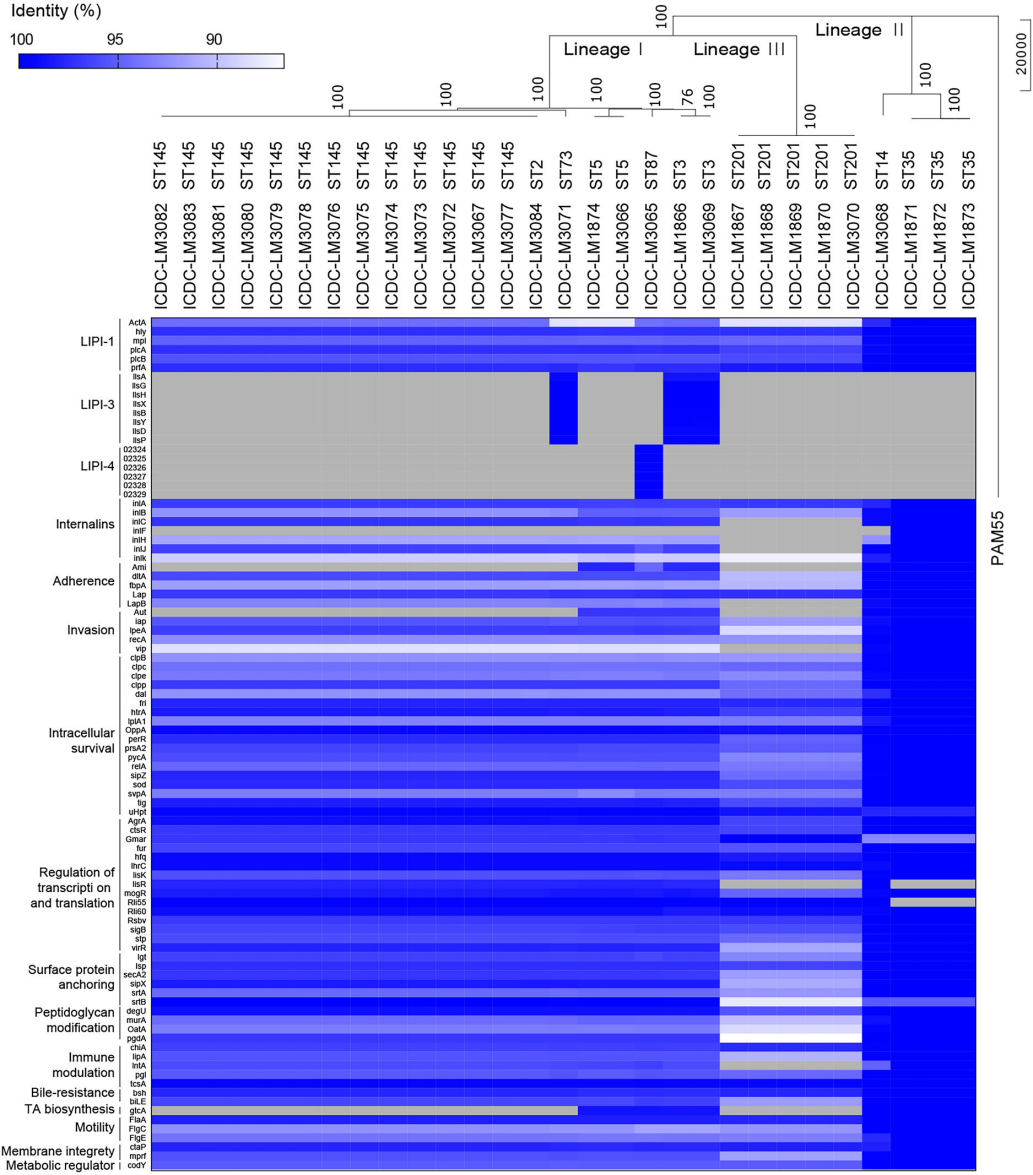
Virulence profiles across the phylogeny of 29 isolates from Dianchi Lake. The phylogenetic analysis based on SNP of core genes using Neighbor-joining method with 1000 bootstrap replicates implemented in the MEGA, and PAM55 (*L. ivanovii*) was used as outgroup. The presence (colour line)/ absence (grey) gene matrix represents, from left to right, genes comprised of LIPI-1 (*prfA, plcA, plcB, hly, mpl, atcA*), LIPI-3 (*llsAGHXYDP*), LIPI-4 (*Clip80459_02324* to *Clip80459_02329*) and other genes involved in internalization (*inlABCFHJK*), adherence (*ami, dltA, fbpA, lap, lapB*), invasion (*aut, iap, ipeA, recA, vip*), intracellular survival (*clpB, clpc, clpe, clpp, dal, fri, htrA, lplA1, oppA, perR, prsA2, pycA, relA, sipZ, sod, svpA, tig, uHpt*), regulation of transcription and translation (*agrA, ctsR, gmar, fur, hfq, lhrC, lisK, lisR, mogR, rli55, rli60, rsbv, sigB, stp, virR*), surface protein anchoring (*lgt, lsp, secA2, sipX, srtA, srtB*), peptidoglycan modification (*degU, murA, oatA, pgdA*), immune modulation (*chiA, lipA, intA, pgl, tcsA*), bile-resistance (*bsh, bilE*), teichoic acid biosynthesis (*gtcA*), motility (*flaA, flgC, flgE*), membrane integrity (*ctaP, mprF*) and metabolic regulator (*codY*).

The 29 *L. monocytogenes* isolates with 28 from the Dianchi Lake black-headed gulls and 1 from Dianchi Lake environment were chosen for whole genome sequencing. The general genome characteristics were shown in Table 2. Core genome SNPs were used to construct a phylogenetic tree by the Neighbor-joining method, and *Listeria ivanovii* PAM55 was used as outgroup. The 29 *L. monocytogenes* isolates were divided into 3 clusters which are consistent with the three Lineages (I-III) previously defined [25] (Figure 1).

**Table 2.** Genome information of L. monocytogenes isolates from Dianchi lake.

Strain	Sequence type	Pulsotype	Time	Assembly size (bp)	NO. of CDS	Protein coding DNA (%)	G + C content (%)	N50	No. of tRNAs	No. of rRNAs (5S, 16S, 23S)
ICDC-LM1866	ST3	PT351	Mar 2016	3020306	3008	90.37	38.47	518423	56	5, 1, 1
ICDC-LM1867	ST201	PT51	Mar 2016	2952181	3004	90.17	38.66	556870	56	5, 1, 1
ICDC-LM1868	ST201	PT51	Mar 2016	2949970	3000	90.08	38.66	530631	54	5, 1, 1
ICDC-LM1869	ST201	PT51	Mar 2016	2950979	3003	90.2	38.66	556870	56	1, 1, 1
ICDC-LM1870	ST201	PT51	Mar 2016	2950530	3002	90.2	38.66	556870	54	5, 1, 1
ICDC-LM1871	ST35	PT59	Mar 2016	2918246	2951	90.49	38.43	584287	65	5, 1, 1
ICDC-LM1872	ST35	PT59	Mar 2016	2927894	2958	90.44	38.43	584287	59	4, 1, 1
ICDC-LM1873	ST35	PT59	Mar 2016	2916200	2945	90.53	38.43	584287	65	4, 0, 1
ICDC-LM1874	ST5	PT22	Mar 2016	2982693	2974	90.51	38.42	1534255	54	5, 0, 1
ICDC-LM3065	ST87	PT30	Jan 2018	2920082	2907	90.02	38.48	355652	56	5, 1, 1
ICDC-LM3066	ST5	PT369	Jan 2018	2996512	3013	90.51	38.41	508204	55	5, 1, 1
ICDC-LM3067	ST145	PT33	Jan 2018	2922729	2898	90.41	38.43	593832	54	4, 1, 1
ICDC-LM3068	ST14	PT261	Nov 2017	2977883	2994	90.56	38.33	543427	58	4, 1, 1
ICDC-LM3069	ST3	PT23	Nov 2017	2943233	2906	90.34	38.52	518829	56	5, 1, 1
ICDC-LM3070	ST201	PT51	Nov 2017	2946382	3000	90.33	38.66	556870	54	4, 0, 0
ICDC-LM3071	ST73	PT374	Nov 2017	2950638	3001	90.19	38.66	556876	54	5, 1, 1
ICDC-LM3072	ST145	PT33	Nov 2017	2924253	2896	90.24	38.44	331902	58	4, 1, 1
ICDC-LM3073	ST145	PT33	Nov 2017	2919296	2897	90.29	38.44	331688	57	4, 1, 1
ICDC-LM3074	ST145	PT33	Nov 2017	2957596	2924	90.1	38.44	331686	58	4, 1, 1
ICDC-LM3075	ST145	PT33	Nov 2017	2922485	2896	90.28	38.44	331688	54	4, 1, 1
ICDC-LM3076	ST145	PT33	Nov 2017	2919691	2888	90.19	38.44	331688	37	4, 1, 1
ICDC-LM3077	ST145	PT33	Nov 2017	2915558	2896	90.34	38.44	331688	57	4, 1, 0
ICDC-LM3078	ST145	PT33	Nov 2017	2919316	2889	9.32	38.44	331688	54	4, 1, 1
ICDC-LM3079	ST145	PT33	Nov 2017	2919932	2893	90.2	38.44	331688	56	4, 1, 1
ICDC-LM3080	ST145	PT33	Nov 2017	2921040	2894	90.18	38.44	331688	58	4, 1, 1
ICDC-LM3081	ST145	PT33	Nov 2017	2918738	2891	90.22	38.44	331688	56	4, 1, 1
ICDC-LM3082	ST145	PT33	Nov 2017	2922311	2894	90.29	38.44	331401	57	4, 1, 1
ICDC-LM3083	ST145	PT33	Nov 2017	2921878	2895	90.19	38.44	331495	57	4, 1, 1
ICDC-LM3084	ST2	PT34	Nov 2017	2957043	2967	90.49	38.41	339275	67	3, 1, 0

To obtain the highest resolution relationships of isolates within an ST, we further constructed different phylogenetic trees based on the core genome SNPs for the 8 STs (ST2, ST3, ST5, ST14, ST35, ST87, ST145 and ST201) separately. These STs contained multiple isolates in this study and isolates from other regions or countries. We describe the results by grouping STs with potential transmission source for Dianchi Lake black-headed gull isolates.

#### STs that contained isolates from foreign countries

**Figure 2.**
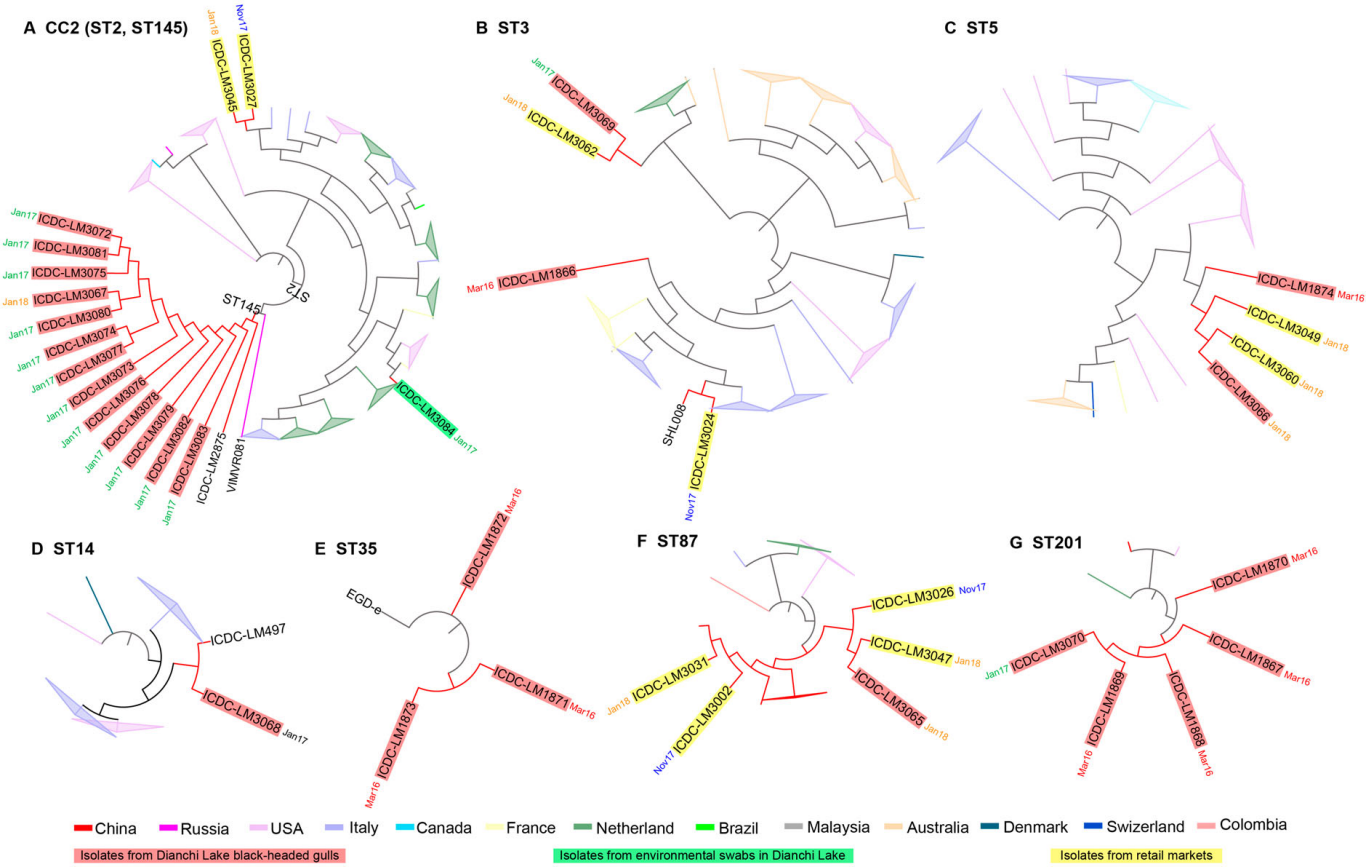
Phylogenetic trees of CCs/ STs based on SNP of core genes. Each CCs/ STs tree was analyzed using Maximum Likelihood method with 1000 bootstrap replicates implemented in the MEGA. (A) CC2 phylogenetic tree, VIMVR081 was used as reference strain. (B) ST3 phylogenetic tree, R2-502 was reference strain. (C) ST5 phylogenetic tree, J2-064 was reference strain. (D) ST14 phylogenetic tree, NRRL B-33805 was reference strain. (E) ST35 phylogenetic tree, EGD-e was reference strain. (F) ST87 phylogenetic tree, ICDC-LM188 was reference strain. (G) ST201 phylogenetic tree, M7 was reference strain. Each colour of node denotes the country which strains comes from. And isolates from black-headed gull feces, environmental swabs in Dianchi Lake and retail markets were shaded in red, green and yellow label background respectively. And the notes beside isolates represent the isolate date. Mar16, March 2016; Jan17, January 2017; Nov17, November 2017; Jan18, January 2018.

ST145 contained 15 isolates including 13 isolates from Dianchi Lake black-headed gulls, and one isolate each isolated from humans in Hebei province of China and Russia respectively. The tree was routed by an ST2 strain. The 13 Dianchi Lake black-headed gull isolates were grouped together and shared a closest relationship with the Hebei isolate with 2–3 SNP differences. The next isolate closest to was a Russian isolate with 8–9 SNP differences (Figure 2A, Table 3).

**Table 3.** The number of SNPs between L. monocytogenes isolates from Dianchi Lake and other regions.

Sequence type (Pulsotype, No. of strains)	Strains of retail markets in Kunming (No. of strains)	Strains of other regions of China (No. of strains)	Strains of foreign countries (No. of strains)
ST2 (PT34, 1, ICDC-LM3084)	123 to130, median 126.6 (2)	–	52–150, median 103 (53)
ST3 (PT351, 1, ICDC-LM1866)	98–117, median 107.5 (2)	93 (1)	74–170, median 93.5 (38)
ST3 (PT23, 1, ICDC-LM3069)	62–141, median 101.5 (2)	136 (1)	93–189, median 122 (38)
ST5 (PT22, 1, ICDC-LM1874)	83, median 83 (2)	–	58–233, median 119 (90)
ST5 (PT369,1, ICDC-LM3066)	8–9, median 8.5 (2)	–	89–262, median 150 (90)
ST14 (PT261, 1, ICDC-LM3068)	–	26 (1)	33–98, median 78 (13)
ST35 (PT59, 3)	–	–	4–11, median 8 (1)
ST87 (PT30, 1, ICDC-LM3065)	25–35, median 29 (4)	26–32, median 23.5 (4)	28–58, median 32 (16)
ST145 (PT33, 13)	–	2–3, median 2 (1)	8–9, median 8 (1)
ST201 (PT51, 5)	–	15–19, median 16 (1)	8–14, median 10.5 (2)

ST201 contained 5 isolates from Dianchi Lake black-headed gulls and 1 isolate from other parts of china and 2 isolates from other countries. The black-headed gull isolates were more closely related to isolates from other countries (The Netherlands and USA) with 8–14 SNP differences than to another China isolate with 15–19 SNP differences (Figure 2G, Table 3).

#### STs that contained isolates from local markets

ST5 contained 94 isolates (including 2 isolates from Dianchi Lake black-headed gulls, 2 isolates from Kunming local retail markets, and 90 isolates from other regions). The two black-headed gull isolates and the two local market isolates were grouped together as one cluster and the smallest SNP difference between black-headed gull isolates and the local market isolates were only 8 SNPs. The Dianchi Lake black-headed gull isolates were separated from isolates from other countries by 58–262 (median, 126) SNPs (Figure 2C, Table 3).

ST87 contained 25 isolates (including 1 isolate from a Dianchi Lake black-headed gull, 4 isolates from Kunming local retail markets, and 20 isolates from other regions). All isolates from China were on one clade. The Dianchi Lake black-headed gull isolate was closest to local market isolates and shared 25–35 (median, 29) SNPs with isolates from retail markets and 26–32 (median, 23.5) SNPs with isolates from other regions of China respectively. Moreover, other isolates from four countries were grouped into 4 independent clades (Figure 2F, Table 3).

#### Other STs

ST3 contained 43 isolates (including 2 isolates from Dianchi Lake black-headed gulls, 2 isolates from Kunming local retail markets, and 39 isolates from other regions). The two Dianchi Lake black-headed gull isolates, ICDC-LM3069 and ICDC-LM1866 were distributed in two clades, ICDC-LM3069 was grouped together with local market isolate ICDC-LM3062 with 62 SNP differences, while ICDC-LM1866 stand by its own on the phylogenetic tree (Figure 2B, Table 3).

ST14 comprised of 15 isolates (including 1 isolates from a Dianchi Lake black-headed gull and 14 isolates from other regions). Dianchi Lake isolate ICDC-LM3068 was grouped together with a Xingjiang food isolate ICDC-LM497 with 26 SNP differences while isolates from different countries were well separated from them. (Figure 2D, Table 3).

ST35 had 4 isolates (3 isolates from Dianchi Lake black-headed gulls and a reference strain EGD-e from Europe). The four isolates were closely related to each other and share 11 SNPs (Figure 2E, Table 3).

ST2 contained 56 isolates (including 1 isolate from Dianchi Lake environment, 2 isolates from Kunming local retail markets, and 53 isolates from other regions) which formed multiple clades. The Dianchi Lake environmental isolate ICDC-LM3084 was closer to an isolate from France with 52 SNP differences than to the two isolates from the local markets with 123–130 SNP differences which were also well separated on the tree (Figure 2A, Table 3).

### Virulence genes identification of *L. monocytogenes* isolates from Dianchi Lake black-headed gulls

All isolates from Dianchi Lake were positive for LIPI-1, and contained the majority of other virulence genes, and each ST has similar virulence profiles. LIPI-3 was found in ST3 and ST73 while LIPI-4 was only found in ST87 (Figure 1).

## Discussion

**Figure 3.**
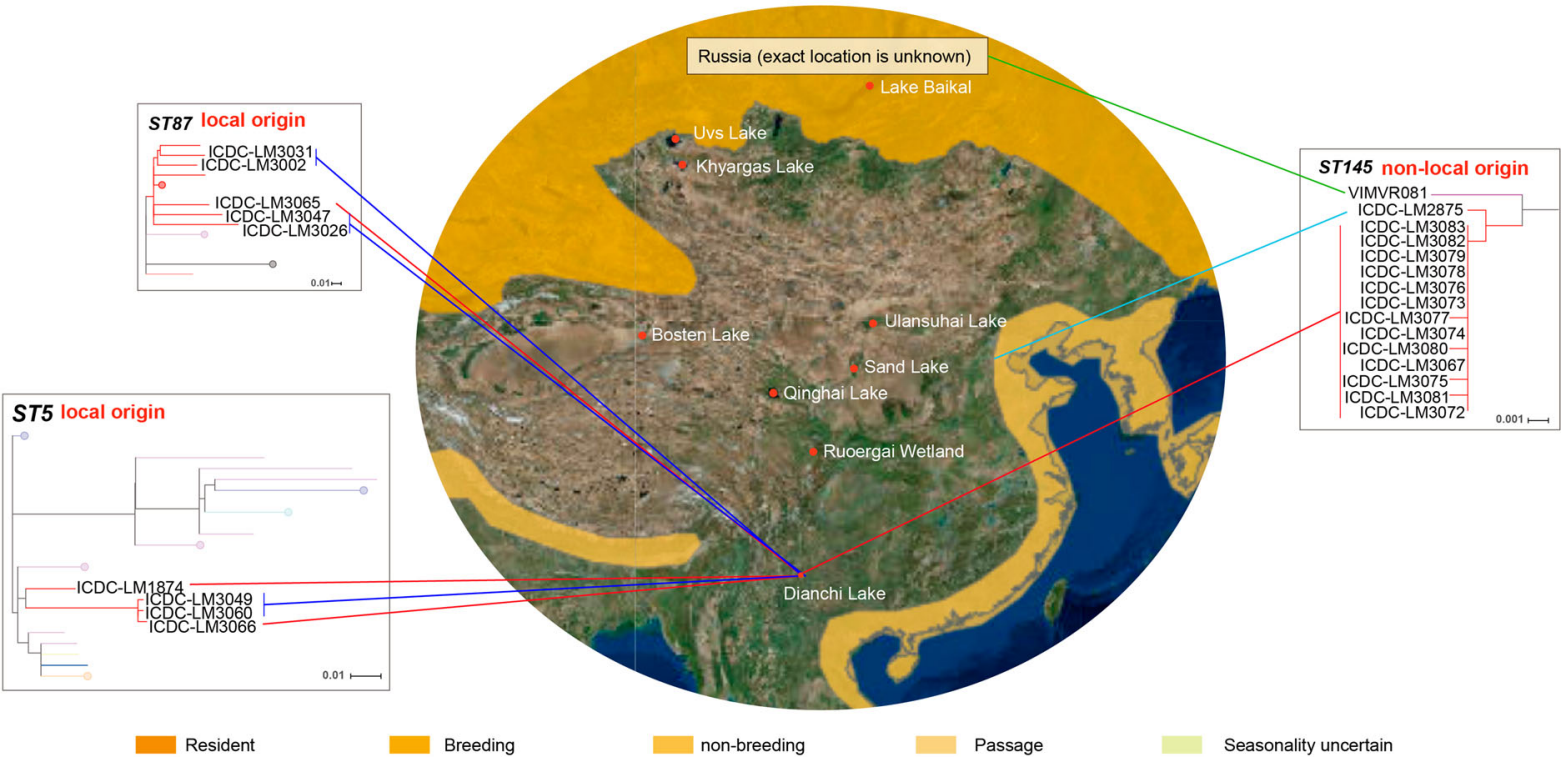
Migratory map of black-headed gulls from Kunming. The migratory information comes from International Union for Conservation of Nature, IUCN (https://www.iucnredlist.org/species/22694420/132548687#assessment-information) and Kunming Institute of Zoology, Chinese academy of sciences (http://www.kiz.cas.cn/xwzx/news5/201706/t20170623_4817599.html). Different shades of yellow on geographic range denote the black-headed gulls’ scope of activity. Phylogenetic trees of ST5, ST87 and ST145 were analyzed using Maximum Likelihood method with 1000 bootstrap replicates implemented in the MEGA. Each colour of node denotes the country which strains comes from, and the colours are consistent with Figure 2. The red line represents the fecal isolates which isolated from Dianchi Lake black-headed gulls; the mazarine line represents the food isolates which isolated from the retail markets proximal to Dianchi Lake; the green line represents the Russian isolate; the wathet line represents the clinical isolate which isolated from Hebei province.

Migratory birds are susceptible to various pathogens such as *Avian influenza virus, Salmonella* and *Campylobacter* [8,15–18,26]. Some wild water birds like black-headed gull could be the ultimate source of *Influenza A virus* and natural reservoir of potentially other pathogens, and spread the pathogens to poultry, mammals and humans [18,27]. In China, black-headed gulls migrate from Xinjiang, Mongolia and Siberia to Dianchi Lake of Kunming every year (Figure 3). And the gulls usually perch at the beach of Dianchi Lake from November to March, there are abundant opportunities for the birds to contact with local residents and tourists. *L. monocytogenes* had been isolated from black-headed gull feces in Dianchi Lake previously [19]. However, the relationship between the source of the *L. monocytogenes* isolates and the wide habitats of migratory gulls is unclear. To address these questions, we carried the surveillance on *L. monocytogenes* in black-headed gulls in Dianchi Lake for three years.

The prevalence of *L. monocytogenes* in black-headed gull feces averaged 0.87% with 1.0%, 1.0% and 0.6% per year from 2016 to 2018, and the occurrences in environmental samples of Dianchi Lake were 0% except in 2017 with 0.7% [19]. The occurrences of *L. monocytogenes* in black-headed gull feces were similar to the result of a recent study of pathogen carriage by migratory birds along the Mediterranean- Black Sea Flyway at 0.6% of *L. monocytogenes* [28]. Of the 28 *L. monocytogenes* isolates obtained from black-headed gulls, 50% of the isolates were serotype 4b which is known to be responsible for the majority of listeriosis outbreaks reported [11]. All *L. monocytogenes* isolates from Dianchi Lake black-headed gulls contain LIPI-1 and *inlA/B*. Further ST3 and ST73 isolates carried LIPI-3 while ST87 isolates carried hypervirulent LIPI-4. Most isolates contained other virulence-related genes which are shown in Figure 1. Although the incidence of *L. monocytogenes* was relatively low, the isolates carried by the gulls might be a potential risk to cause listeriosis in humans, and our 3 years of surveillance showed that *L. monocytogenes* carriage by black-headed gulls was an ongoing problem.

*L. monocytogenes* is a foodborne bacterium which is widely distributed in foods and food processing environments. Thus food products and environmental conditions would be reflective of the local epidemiology of *L. monocytogenes*. In this study, the prevalence of *L. monocytogenes* in retail markets near Dianchi Lake (16.49%) was close to the previous study in Kunming (16.2%) and the isolates share similar subtypes with others studies on Chinese food retail markets [14,29–31].

In this study, we also surveyed retail markets for *L. monocytogenes* to determine whether there was any link between *L. monocytogenes* isolates from Dianchi Lake black-headed gulls and local isolates and possible inter-transmission of *L. monocytogenes* between the gulls and local environment. The predominant subtypes of *L. monocytogenes* from black-headed gulls and retail markets were completely different, however, they shared some STs and/or PTs including ST3, ST5 (PT22) and ST87 (PT30). For ST5 and ST87, isolation rate from local food source was 7.8% and 28.1% of food isolates respectively, similar to other studies in China [14,30]. These STs were frequently isolates from food sources and ST87 is the predominant ST causing human listeriosis in China [32,33], pointing to potential local source of some STs of *L. monocytogenes* from the black-headed gulls.

Genome sequencing offered a higher resolution to address these questions. However, it should be noted that it is difficult to use a SNP cutoff to determine the epidemiological link of isolates in *Listeria*. Isolates of the same origin years apart may differ by very few SNPs while isolates from the same outbreak may differ by up to 50 SNPs [34]. Reimer *et al* analyzed 9 outbreaks and showed that epidemiologically confirmed outbreak isolates can differ by as much as 30 SNPs [35]. The mutation rate of *L. monocytogenes* was reported to be 0.4 SNPs per genome per year [21], which cannot be used to easily reconcile these observations. Nevertheless, we used 3 criteria to assess the likely origin of the *L. monocytogenes* from the black-headed gulls: (1) number of SNP differences between isolates of different sources; (2) comparison with local retail market isolates, isolates from other parts of China and isolates from other countries; and (3) regional prevalence of different STs. The inference of origin doesn’t suggest a direct epidemiological link of the same point source, for example, from the retail market where we sampled.

For ST5, the 2 gull isolates and the food isolates were grouped together, with the 2016 gull isolate diverged earlier. The 2018 gull isolate differed from one of the 2018 local market isolate by only 8 SNPs. For the ST87 isolates, the gull isolate is also closest to a local market isolate from the same year with 25 SNP differences. These two STs were likely to be sourced locally.

The source of the ST145 isolates carried by black-headed gulls was likely to be non-local and possibly from its breeding location in its origin. ST145 belongs to serotype 4b and was rarely reported worldwide. Isolates from Dianchi Lake black-headed gulls were closely related to an isolate from Hebei in the north of China and isolates from Russia. ST145 has never been reported to cause disease in China before, there is no more case reported in Hebei so far, and the transmission pathway for the only isolate from Hebei was unknown due to the lack of epidemiological information and there were no more ST145 isolates reported. In contrast, ST145 strains have been reported in river waters and rodents in southern Russia [36–38]. It is likely that the black-headed gulls carried ST145 *L. monocytogenes* from another location like southern Russia to Dianchi Lake. Further studies will be required to confirm this inference using tracking data. For ST201, the source was also likely to be non-local. The 5 gull isolates were closer to each other and closer to isolates from other countries with 8 SNP differences. The ST wasn’t sampled from the local market and was rare in China [39,40].

For ST3, ST14, and ST35 isolates, the source is unclear as no closely related local or foreign isolates were found. The Dianchi Lake black-headed gull ST3 isolate (ICDC-LM3069) was grouped with a local market isolate with 62 SNP differences while the ST14 isolate (ICDC-LM3068) was grouped with a food-source isolate from Xinjiang with 26 SNP differences. It is indeterminant the source of the ST3 isolates. However, for the ST14 isolate, it is possible that black-headed gull acquired the ST14 isolate in Xinjiang which was known to be a stopover locale for black-headed gull migration.

It is also interesting to note that isolates from black-headed gull from different years may be closer to each other than with other sources (for example ST145 isolates), suggesting that the same *L. monocytogenes* population may be maintained in the black-headed gull population or the black-headed gulls may obtain *L. monocytogenes* from the same source in different years. Both scenarios are possible considering that black-headed gulls are territorial with lifespan of up to 30 years and may pass through the same locale every year [41]. It has been shown that *L. monocytogenes* can persist in birds for long periods of time by carriage [42]. It is also possible that there are other sources of *L. monocytogenes* such as the other avian species which transmit the pathogen through the common environment. Further studies are required to address these questions.

Our results suggest that black-headed gulls could facilitate *L. monocytogenes* spread across geographical regions through migration, concurring with previous studies showing that pathogens could spread to another geographically distant sites through migratory birds [43,44]. Although the prevalence of *L. monocytogenes* in black-headed gulls was low, our findings underscore the importance of surveillance of *L. monocytogenes* in the migratory black-headed gulls, which would be helpful to reduce the infection risk to humans in regions where the migratory birds reside.

## Supplementary Material

Supplemental Material
