# Supplementary figures and images for: Carriage and potential long distance transmission of Listeria monocytogenes by migratory black-headed gulls in Dianchi Lake, Kunming
Source: Emerg Microbes Infect. 2019 Aug 8;8(1):1195–204. doi: 10.1080/22221751.2019.1647764 (PMC6713206; doi:10.1080/22221751.2019.1647764)

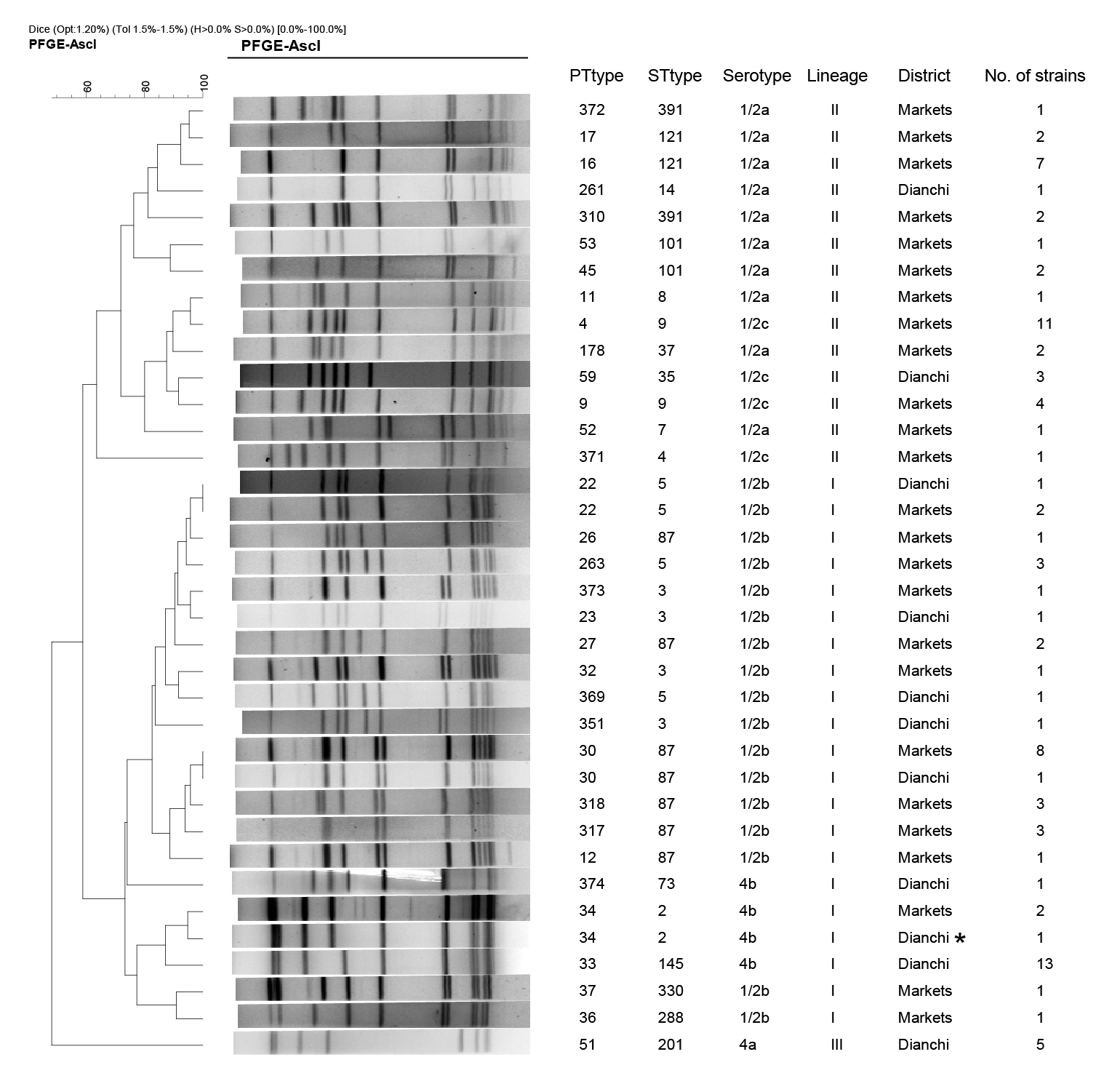

Supplement: Supplemental Material [file TEMI_A_1647764_SM8334.zip › supplemental figure1.jpg]
